# Microwave-Induced Structural Remodeling of Legume Proteins: Structure–Function–Nutrition Relationships and Their Improved Performance in Wheat Flour Fortification

**DOI:** 10.3390/foods15030580

**Published:** 2026-02-05

**Authors:** Nikhil Dnyaneshwar Patil, Prabhat Kumar, Aarti Bains, Minaxi Sharma, Kandi Sridhar, Prince Chawla, Baskaran Stephen Inbaraj

**Affiliations:** 1 Department of Food Technology and Nutrition, Lovely Professional University, Phagwara 144411, India; 2 Coconut Development Board Quality Testing Laboratory, Ministry of Agriculture & Farmers Welfare, Government of India, Kochi 683105, India; 3 School of Health Sciences and Technology, University of Petroleum and Energy Studies, Dehradun 248007, India; 4 Research Centre for Life Science and Healthcare, Nottingham Ningbo China Beacons of Excellence Research and Innovation Institute (CBI), University of Nottingham Ningbo China, Ningbo 315104, China; 5 Department of Food Technology, Karpagam Academy of Higher Education (Deemed to be University), Coimbatore 641021, India; 6 Department of Food Science, Fu Jen Catholic University, New Taipei City 24205, Taiwan

**Keywords:** microwave-assisted extraction, legume protein, functional properties, in vitro digestibility, amino acid content

## Abstract

The study explored the impact of Microwave-Assisted Extraction (MAE) on the physicochemical, structural, functional, and antioxidant properties of protein concentrates from white pea (*Lathyrus sativus*), red gram (*Cajanus cajan*), and black gram (*Vigna mungo*). The objective was to evaluate the efficiency of MAE as a sustainable green extraction technique compared to the conventional method. Total amino acid content increased in MAE protein from 69.23 to 72.78 g/100 g powder in white pea protein (WPP), 69.41 to 72.39 g/100 g powder in red gram protein (RGP), and 65.56 to 70.30 g/100 g powder in black gram protein (BGP). Functionally, MAE significantly improved solubility and emulsifying capacity and water- and oil-holding capacities. Bioactive evaluation showed a significant increase in total phenolic and flavonoid contents, followed by improved DPPH, ABTS, and FRAP activities. A reduction in tannins and phytic acid correlated with enhanced in vitro protein digestibility. These enhanced MAE-derived proteins further demonstrated superior performance when incorporated into wheat flour, improving its nutritional and functional properties. Overall, MAE protein demonstrated improved structural integrity, antioxidant potential, and digestibility, highlighting white pea protein as the most responsive legume to MAE, followed by red and black gram.

## 1. Introduction

The rapid growth of the global population, along with increasing pressures on traditional animal farming, has created an urgent need for sustainable, high-quality protein sources to ensure both nutritional security and environmental stability [1]. Plant-based proteins, especially those from legumes, have emerged as promising alternatives, offering a way to meet protein demands with minimal environmental impact [2]. In recent years, environmentally friendly extraction techniques have gained attention, emphasizing the use of low solvents, energy, and water, improving environmental sustainability [3]. Modern approaches, such as enzyme-assisted processes, subcritical water extraction, ultrasound-assisted extraction, and pulse electric field extraction, are transforming protein recovery by making it more sustainable and cost-effective [4]. However, balancing high extraction efficiency with the preservation of protein functionality remains a key challenge, driving ongoing research and innovation in sustainable protein production.

Traditional extraction methods have long been used to isolate proteins from plants and legumes. Common approaches include alkaline and acid extraction, isoelectric precipitation, and salt solubilization, which rely on high-pH or -salt solutions to release proteins from complex plant matrices [5]. Traditional wet extraction methods have several limitations, including the presence of excess solvent residues, environmental problems, and high energy use. These methods often give low protein yields and poor-quality extracts. Alkaline extraction can reduce protein digestibility, change amino acid structures, and create a bitter taste. Acid extraction also affects the solubility and gel-forming properties of proteins. Dry fractionation methods, like air classification, leave some protein in the starch portion, which lowers the total protein yield. In many conventional processes, large amounts of volatile, flammable, and toxic solvents are used, and some of these solvents may remain on the protein and pose health risks. Because of these issues, researchers are working on improved extraction methods to overcome these drawbacks [6,7]. Therefore, despite their long-standing use, conventional extraction techniques face significant challenges in environmental efficiency and protein quality, highlighting the need for alternative approaches that provide both sustainability and high-quality protein isolates.

To address the limitations imposed by conventional extraction, this study has adopted microwave-assisted extraction (MAE) as an innovative green approach for isolating protein from alternative legumes. Microwaves generate non-ionizing electromagnetic waves. They heat a sample through dipole rotation and ionic conduction, which disrupts hydrogen bonds within the plant cell wall. This disruption increases cell wall porosity, allowing for better solvent penetration and enhancing the release of intracellular compounds. As microwave energy is converted into heat within the matrix, rapid moisture evaporation generates internal pressure that weakens the cell wall. MAE can be performed in open or closed vessels depending on the required temperature and pressure. Open systems are used under ambient conditions, while closed systems are suitable for high-temperature and high-pressure extraction [8,9]. Studies have shown that microwave-assisted extraction improves protein recovery across a wide range of plant and microbial sources. The technique enhances cell disruption, accelerates solvent penetration, and promotes faster release of intracellular components. Compared with conventional extraction methods, microwave treatment delivers higher yields, shorter processing times, and more efficient mass transfer, demonstrating its strong potential for sustainable protein extraction [10,11,12,13,14,15]. In addition, MAE offers advantages over conventional thermal extraction, including uniform heating, faster extraction, lower solvent usage, and reduced processing time [16].

This study focuses on three underutilized but nutritionally valuable legumes: white pea (*Lathyrus sativus*), red gram (*Cajanus cajan*), and black gram (*Vigna mungo*). These pulses were selected for their high protein content, balanced amino acid profiles, and functional properties. Black gram is a good source of protein, dietary fiber and essential minerals [17]. Red gram provides high-quality protein, essential amino acids, and water-soluble vitamins, making it a valuable nutritional ingredient. In addition, red gram contains a wide range of bioactive compounds that contribute to anti-inflammatory, antibacterial, antioxidant, anticarcinogenic, and antidiabetic activities [18]. White pea is a nutrient-dense cool-season legume belonging to the Fabaceae family, characterized by its high protein content (20–25%) and substantial levels of essential minerals and vitamins [19]. Despite its nutritional strength and use in traditional foods like dal, vada, or idli, black gram and red gram protein ingredients are not widely available on the market, largely because they are used mostly in whole or split grain forms and face less investment in innovative protein isolation technologies compared to soya and chickpea.

Most studies on legume protein extraction have focused on conventional methods, showing variable yields, structural stability, and functional properties. Research on microwave-assisted extraction (MAE) remains limited, particularly regarding its effects on amino acid composition, protein structure, functional behavior, antioxidant activity, and in vitro digestibility. This study aimed to evaluate the effects of microwave-assisted extraction (MAE) on the white pea protein (*Lathyrus sativus*), red gram protein (*Cajanus cajan*), and black gram protein (*Vigna mungo*) compared to conventional methods. MAE’s impact on protein recovery, amino acid composition, and structural characteristics was evaluated, while functional properties, including solubility, water- and oil-holding capacity, emulsifying activity, and foaming ability were analytically measured. Antioxidant potential was assessed using multiple assays, and in vitro protein digestibility was determined. By integrating compositional, structural, and functional evidence, the study demonstrates that microwave-assisted extraction favorably modifies key protein quality attributes, including recovery, functional performance, and nutritional accessibility, without compromising structural integrity. These combined effects indicate that MAE represents an efficient and environmentally sustainable approach for the extraction of high-quality plant proteins.

## 2. Materials and Methods

### 2.1. Materials

Red gram (*Cajanus cajan*), black gram (*Vigna mungo*), and white pea (*Pisum sativum*) seeds were obtained in 2023 from the Indian Institute of Pulses Research (IIPR), Kanpur, Uttar Pradesh, India. All chemicals and reagents used in the present study were of analytical grade to maintain precision and reliability throughout the experiments. Analytical procedures were carried out following the standard protocols outlined by AOAC and other well-established scientific practices.

### 2.2. Methods

#### 2.2.1. Proximate Composition of Different Legumes

The proximate composition of white pea, red gram, and black gram powder was determined using the official methods described by Hossain et al. [20]. The analysis included moisture, crude protein, ash, crude fiber, and crude fat. Protein content was estimated by the micro-Kjeldahl technique (FOSS India Pvt. Ltd., Pune, India), crude fiber was assessed through sequential acid–alkali digestion (FOSS India Pvt. Ltd., Pune, India), and fat was quantified using a Soxhlet extraction system. Carbohydrate content was calculated by difference, subtracting the percentages of protein, moisture, ash, and fat from 100%, with adjustments made according to standard conversion factors.

#### 2.2.2. Microwave-Assisted Extraction of Protein

Protein isolation from legume seeds was performed following the method of Bhatnagar et al. [21] with slight modifications. Briefly, 200 g of seeds were soaked in deionized water at a seed-to-water ratio of 1:7 (*w*/*v*) for 12 h. The hydrated seeds were ground using a mixer grinder (MX-AC400, Panasonic, Gurugram, India) with deionized water (1:6, *w*/*v*) to obtain a slurry, which was stirred magnetically (Remi 2MLH, Remi Equipments Pvt. Ltd., Mumbai, India) at 800 rpm for 15 min at 27 ± 2 °C. The pH of the slurry was adjusted to 11 using 1 M NaOH and maintained under continuous stirring for 30 min at the same temperature. For microwave-assisted extraction (MAE), 100 mL of the alkaline slurry was transferred into a 250 mL borosilicate glass beaker and treated in a convection microwave oven (LG 32 L, model MC3286BLU, LG Electronics, Noida, India) operating at 2450 MHz. Microwave treatment was conducted at power levels ranging from 180 to 720 W for exposure times of 30–120 s, as specified in Table 1. The slurry volume and container size were kept constant for all treatments to ensure uniform energy absorption. The final temperature of the slurry was measured immediately after microwave treatment using a digital probe thermometer (TP-101, Ravi Scientific Industries, Delhi, India) and ranged from 34 to 100 °C, depending on the applied power level and exposure time (Table 1). Native legume protein was prepared using the same alkaline extraction conditions, including identical pH adjustment (pH 11), stirring rate (800 rpm), extraction time (30 min), and temperature (27 ± 2 °C), but without microwave treatment. Following extraction, the slurry was centrifuged at 6000 rpm for 30 min (Remi 8C Plus, Remi Equipments Pvt. Ltd., Mumbai, India), and the supernatant was collected. The residual pellet was re-suspended in deionized water (1:8, *w*/*v*), stirred at 500 rpm for 30 min, and centrifuged again under identical conditions. Protein precipitation was achieved by adjusting the combined supernatant to pH 4.5 using 1 M HCl, followed by centrifugation at 6000 rpm for 30 min. The precipitated protein was washed with deionized water and centrifuged again to remove residual impurities. The recovered protein fraction was dissolved in deionized water, neutralized to pH 7 using 1 M NaOH under gentle stirring (500 rpm), and subsequently dried in tray dryer (Lablink Pvt Ltd, Thane, India) at 45 °C to obtained powder form. The protein powder was then stored in airtight containers at 4 °C until further analysis. Proteins obtained through alkaline extraction without microwave treatment were designated as native proteins (NBGP, NWPP, and NRGP). In this study, the alkaline-extracted protein obtained without microwave treatment was considered a native protein. The alkaline-extracted protein obtained through this process showed protein contents of 68.74 ± 0.32% for black gram (NBGP), 72.26 ± 0.32% for white pea (NWPP), and 71.11 ± 0.40% for red gram (NRGP).

#### 2.2.3. Protein Content

The protein concentration was estimated using the colorimetric method described by Lowry et al. [22], with slight modifications.

#### 2.2.4. Functional Properties of Protein

##### Protein Solubility

Protein solubility was assessed following the procedure of Zeng et al. [23] with slight modifications. For this analysis, 1 g of the sample was suspended in 50 mL of deionized water and stirred magnetically for 1 h. The mixture was then centrifuged at 6000 rpm for 30 min, after which the supernatant containing the soluble fraction was collected. Protein concentration in the supernatant was quantified using the Lowry assay, and solubility (%) was calculated using Equation (1).

(1) $$\mathrm{Protein}\;\mathrm{solubility}=\left(\frac{PCS}{TPCS}\right)\times 100$$

In this equation, PCS denotes the protein concentration present in the supernatant, whereas TPCS refers to the total protein content of the initial sample.

##### Water- and Oil-Holding Capacity of Protein (WHC and OHC)

The water- and oil-holding capacities of protein powder were determined following the procedure of Ma et al. [24] with slight modifications. Pre-weighed centrifuge tubes (50 mL) were each loaded with 2 g of protein powder and mixed separately with 20 mL of distilled water or soybean oil. Samples were left undisturbed for 30 min and subsequently centrifuged at 10,000 rpm for 15 min. The supernatant was discarded, and the tubes were weighed again. Water- and oil-holding capacities were expressed as grams of liquid retained per gram of sample (g/g).

##### Foaming Capacity and Stability (FC and FS)

Foaming properties of the protein powder, including foaming capacity (FC) and foam stability (FS), were evaluated according to the method of Liang et al. [25] with minor modifications. Briefly, 1 g of protein sample was dispersed in 50 mL of distilled water and homogenized at 10,000 rpm for 10 min. The whipped solution was immediately transferred into a graduated cylinder, and the initial volume was recorded as V1 (mL). After standing undisturbed for 30 min, the foam volume was measured again as V2 (mL). FC and FS were calculated using Equations (2) and (3).

(2) $$\mathrm{FC}\;(\%)=\left(\frac{V1-50}{50}\right)\times 100$$

(3) $$\mathrm{FS}\;(\%)=\left(\frac{V2-50}{50}\right)\times 100$$

##### Emulsion Capacity and Stability (EC and ES)

The emulsifying properties of the protein samples were assessed following the method of Lawal et al. [26] with slight modifications. Emulsions were prepared by blending 5 mL of a 2% (*w*/*v*) protein dispersion with 5 mL of canola oil using a homogenizer (RQT-127, Remi Equipment Pvt Ltd, Mumbai, India). The mixtures were centrifuged at 12,000 rpm for 5 min to promote phase separation. Emulsion activity (EA) was evaluated by recording the height of the emulsified layer relative to the total sample height in the centrifuge tube, and the value was expressed using Equations (4) and (5).

(4) $$\mathrm{EC}\;(\%)=\left(\frac{E}{T}\right)\times 100$$

In this equation, *E* indicates the measured emulsion layer height, whereas *T* represents the total column height of the mixture in the tube.

For emulsion stability assessment, the prepared emulsions were incubated at 80 °C for 30 min, followed by centrifugation at 6000 rpm for 5 min. Emulsion stability (ES) was calculated using Equation (5).

(5) $$\mathrm{EC}\;(\%)=\left(\frac{E}{T}\right)\times 100$$

Here, *E* indicates the height of the emulsion layer after treatment, while *T* refers to the overall height of the sample in the tube.

#### 2.2.5. Characterization of Protein

##### Amino Acid Content

Amino acid profiling of protein followed the method according to Patil et al. [27]. For hydrolysis, nearly 10 mg of the protein sample was placed in a vial with 20 mL of 5 M HCl containing a few phenol crystals. Following hydrolysis, the mixture was dried under vacuum conditions at 110 °C for a period of 25 h. This hydrolysate was used for quantifying all amino acids except tryptophan. Derivatization was carried out by mixing the hydrolyzed samples with borate buffer (pH 8.2–10) and treating them with the AccQ-Fluor reagent kit (WAT052890, Waters Millipore, Milford, MA, USA), as recommended by the supplier. Amino acid separation and detection were achieved by binary gradient HPLC equipped with a fluorescence detector (HPLC-FLD) using an AccQ-Tag column. The amino acid content was quantified by constructing a calibration curve (0.02–0.5 µmol L^−1^) based on 16 reference amino acid standards.

##### Fourier Transform Infrared Spectroscopy (FTIR)

The identification of functional groups was performed using an FTIR spectrometer (Perkin Elmer X400, PerkinElmer Inc., Shelton, CT, USA) in line with the methodology described by Sharma et al. [28].

##### Thermogravimetric Analysis (TGA)

The thermal stability and mass loss characteristics of the protein powders were examined through TGA on a PerkinElmer Pyris 1 analyzer (PerkinElmer Inc., Shelton, CT, USA), applying the procedure outlined by Sharma et al. [28].

##### Scanning Electron Microscope (SEM)

The morphological characteristics of the protein powder were analyzed using a Field Emission Scanning Electron Microscope (FE-SEM; JEOL JSM-7610F Plus, JEOL Ltd., Tokyo, Japan) equipped with an energy-dispersive X-ray spectroscopy detector (EDS; Oxford Instruments, Oxford, UK; LN_2_-free), following the method described by Sharma et al. [28]. 

#### 2.2.6. Bioactive Properties of Protein

##### Total Phenolic and Flavonoid Content

Determination of phenolic and flavonoid levels was conducted in accordance with the methodology of Roshanak et al. [29]. For the extraction of bioactive constituents, the sample powder was homogenized with 70% aqueous ethanol (100 mL) at 10 °C and agitated continuously at 100 rpm for approximately 16 h. Following extraction, the suspension was clarified by centrifugation at 3000 rpm for 10 min at 4 °C. This extraction cycle was conducted twice more to ensure maximum recovery of phenolic compounds. All collected supernatants were combined, passed through Whatman No. 1 filter paper to eliminate particulate matter, and subsequently concentrated via freeze-drying. The dried extract was preserved at −20 °C until chemical assessment. The phenolic constituents in the extract were quantified using the Folin–Ciocalteu colorimetric assay in the presence of sodium carbonate, with results expressed as mg gallic acid equivalents (GAE) per g of dry extract. Flavonoids were estimated using an aluminum chloride complexation method, and the concentrations were expressed as quercetin equivalents (QE).

##### DPPH Radical Scavenging Activity

Assessment of antioxidant activity through DPPH radical scavenging was performed in line with the protocol of Sharma et al. [30]. The antioxidant potential was assessed using a DPPH radical scavenging assay. Briefly, the extract was reacted with a 0.1 mM solution of DPPH, and the mixture was maintained at 37 °C for 30 min in complete darkness to avoid the photodegradation of the radical. After incubation, the decrease in absorbance at 517 nm was measured using UV-visible spectrophotometer (Systronics-168, Systronics India Ltd., Ahmedabad, India). Control samples contained all reagents except the extract powder. The DPPH radical scavenging activity was calculated as a percentage inhibition using Equation (6).

(6) $$\mathrm{DPPH}\;\mathrm{scavenging}\;(\%)=\left(\frac{Ac-As}{Ac}\right)\times 100$$

Here, *Ac* indicates the absorbance of the control, while *As* refers to the absorbance of the sample.

##### ABTS Radical Scavenging Activity

Measurement of ABTS radical scavenging potential was performed in line with the protocol reported by Hussen et al. [31]. The ABTS assay was employed to evaluate the extract’s ability to neutralize the ABTS^+^ radical cation. The radical solution was generated by combining ABTS (7 mM) with potassium persulfate (2.4 mM) and allowing the reaction to proceed until a stable blue-green chromophore was formed. Then, 40 µL of the sample extract was added to 2.0 mL of the ABTS^+^ working solution, and the mixture was allowed to stand for 15 min at room temperature. The reduction in absorbance at 734 nm was recorded, and scavenging activity was calculated by following Equation (7).

(7) $$\mathrm{ABTS}\;\mathrm{scavenging}\;(\%)=\left(\frac{A0-A1}{A0}\right)\times 100$$

Here, *A*0 indicates the absorbance of the control, while *A*1 refers to the absorbance of the sample.

##### Ferric Reducing Power Assay

Assessment of ferric reducing antioxidant potential of the extract was carried out in accordance with the protocol outlined by Gulcin et al. [32]. The reducing capability of the extract was examined using the potassium ferricyanide reduction assay. In brief, 100 μL of the sample was combined with 2.5 mL of 1% potassium ferricyanide prepared in 0.2 M sodium phosphate buffer (pH 6.6). The reaction mixture was incubated at 50 °C to facilitate the reduction process, after which 2.5 mL of 10% trichloroacetic acid (TCA) was introduced to terminate the reaction and promote protein precipitation. The resulting solution was clarified, and 2.5 mL of the supernatant was mixed with an equal volume of distilled water, followed by 0.5 mL of 0.1% FeCl_3_. The absorbance of the developed Prussian blue complex was recorded at 700 nm, and the reducing power was presented as mg BHT equivalents (BHTE) per g of dry extract.

##### Tannin and Phytic Acid Content

The estimation of tannins was performed following the protocol of Abera et al. [33], with outcomes expressed in tannic acid equivalents. Phytic acid was quantified using the approach outlined by Abera et al. [33].

#### 2.2.7. In Vitro Digestibility of Protein

The simulated gastrointestinal digestion of protein was performed using a two-stage enzymatic system adapted from established in vitro digestion models as described by Han et al. [34] with minor modifications. Protein dispersions (5 mg/mL; 100 mL) were prepared in deionized water and equilibrated at 37 °C prior to enzyme addition. For the gastric phase, the samples were mixed with pepsin to obtain a final activity of 2000 U/mL, the pH was adjusted to 2.5, and incubation proceeded at 37 °C with continuous agitation for 2 h. After gastric hydrolysis, the mixture was diluted (1:1 *v*/*v*) with simulated intestinal medium containing trypsin (100 U/mL), lipase (2000 U/mL), and bile salts (10 mM). The pH was then neutralized to 7.0, and digestion continued under identical temperature and shaking conditions for an additional 2 h. Aliquots were collected at three time points: prior to enzyme treatment (0 h), after completion of the gastric phase (2 h), and following intestinal digestion (4 h). Enzymatic reactions were halted immediately by rapid cooling on ice, and samples were stored at 4 °C until analysis. Protein digestibility was assessed according to Han et al. [34]. Protein digestibility was quantified by separating TCA-soluble peptides from undigested protein. One milliliter of the digested mixture was combined with an equal volume of 10% (*w*/*v*) trichloroacetic acid to precipitate unhydrolyzed proteins, followed by centrifugation at 10,000× *g* for 15 min at 4 °C. The pellet was washed repeatedly with chilled acetone, dried, and dissolved in 1 M NaOH for protein determination using the Bradford assay. The TCA-soluble fraction was evaluated spectrophotometrically at 280 nm, and peptide concentrations were interpolated from a BSA calibration curve. Digestibility (%) was calculated as the proportion of soluble peptides relative to the total protein content of the sample.

#### 2.2.8. Preparation of Protein Fortified Flour

Whole-wheat flour (WWF) was blended with legume proteins derived from white pea, red gram, and black gram at inclusion levels of 5% and 10%. The mixtures were homogenized using an electric mixer (Model 5K45SS Heavy Duty, KitchenAid, Greenville, OH, USA) for 1 min to ensure uniform distribution of the added proteins Sachanarula et al. [35].

##### Proximate Analysis of Flour and Protein Powder

The proximate composition of the sample was analyzed following the official AOAC methods [36]. The parameters measured included moisture, crude protein, ash, crude fiber, and crude fat. Protein content was determined using the micro-Kjeldahl method, crude fiber was measured through sequential acid–alkali digestion, and fat was quantified via Soxhlet extraction. Carbohydrate content was obtained by difference (100% − [protein + moisture + ash + fat]), using standard conversion factors for each proximate component.

##### Water- and Oil-Holding Capacity of the Flour

The water- and oil-holding capacities of flour were determined following the procedure of Ma et al. [24] with slight modifications. Pre-weighed 50 mL centrifuge tubes were filled with 2 g of flour and mixed with 20 mL of either distilled water or soybean oil. The mixtures were allowed to stand for 30 min and then centrifuged at 10,000 rpm for 15 min. After discarding the supernatant, the tubes were reweighed. Water-holding and oil-holding capacities were calculated as the grams of liquid retained per gram of sample (g/g).

##### Water Solubility Index

The water solubility index (WSI) was measured according to Du et al. [37] with minor modifications. Briefly, 1 g of flour was mixed with 10 mL of distilled water in a 15 mL centrifuge tube and heated in a water bath at 37 °C for 30 min. The mixture was then centrifuged at 4000 rpm for 10 min. The supernatant was carefully transferred to a pre-weighed beaker and dried at 105 °C to constant weight. The dried solid obtained from the supernatant was recorded as the soluble fraction. WSI was expressed as the percentage of the dried soluble material relative to the original flour weight.

$$\mathrm{WSI}\;(\%)=\frac{Weight\;of\;dried\;supernatant}{Dry\;weight\;of\;sample}\times 100$$

#### 2.2.9. Statistical Analysis

Each experiment was repeated three times, and the outcomes are reported as mean ± SD. Statistical comparisons were performed in SPSS software (Version 27, IBM Corp., Armonk, NY, USA) employing one-way ANOVA, two-way ANOVA, independent *t*-test, and Duncan’s multiple range test. Differences were regarded as significant at *p* < 0.05.

## 3. Results

### 3.1. Proximate Analysis of the Red Gram, White Pea, and Black Gram

As shown in Table 2, the proximate composition of red gram, black gram, and white pea revealed substantial differences in their nutritional profile. The moisture content was highest in red gram (10.95 ± 0.35%), followed by black gram (10.77 ± 0.10%), and lowest in white pea (7.56 ± 0.34%), indicating variations in hygroscopicity and potential storage stability. The relatively lower moisture content of white pea suggests better shelf stability compared to the other legumes. Ash content was relatively uniform across the samples, ranging from 3.01 ± 0.04% in red gram to 3.02 ± 0.02% in white pea and 3.17 ± 0.03% in black gram, reflecting similar mineral composition among the three pulses. Fat content varied, with white pea exhibiting the highest value (2.72 ± 0.05%), red gram at 1.21 ± 0.03%, and black gram with the lowest (1.02 ± 0.06%), which may influence both energy density and sensory attributes. In terms of protein content, black gram represents the highest value (26.22 ± 0.31%), followed by white pea (24.74 ± 0.36%) and red gram (23.72 ± 0.40%). This observation aligns with earlier findings indicating that black gram is a rich source of high-quality plant protein and can play a key role in protein supplementation, as reported by Kavitha et al. [38]. Crude fiber content ranged from 6.24 ± 0.06% in red gram, 6.16 ± 0.05% in white pea, to 5.11 ± 0.08% in black gram, suggesting potential digestive benefits, particularly from red gram and white pea. The carbohydrate contents of the legumes were 54.87 ± 0.11% in red gram, 55.80 ± 0.21% in white pea, and 53.53 ± 0.50% in black gram. The values for all three legumes were very similar, and the small variations observed were mainly due to differences in moisture content rather than actual differences in carbohydrate levels. Overall, the high carbohydrate content indicates that these pulses serve as important energy-providing foods.

### 3.2. Protein Content

**Figure 1:**
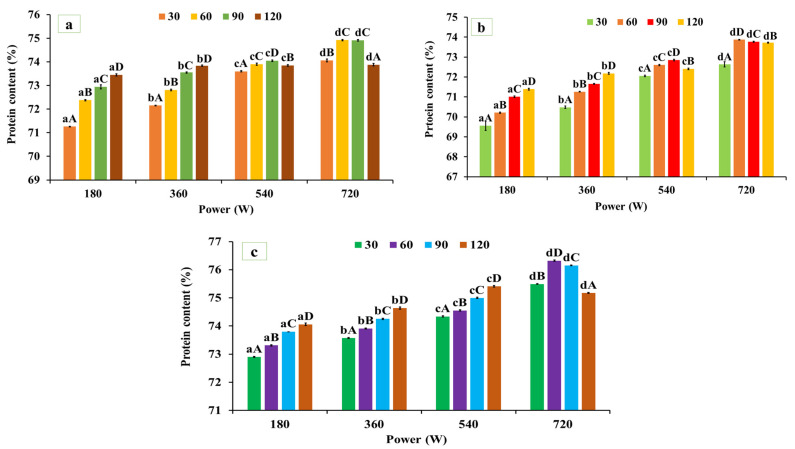
Protein content in the microwave-assisted extracted (**a**) red gram protein, (**b**) black gram protein, and (**c**) white pea protein. Data are presented as mean ± SD (n = 3). Mean values with different lowercase superscripts (a–d) within the same exposure time indicate significant differences among microwave power levels, while mean values with different uppercase superscripts (A–D) within the same power level indicate significant differences among exposure times. Statistical significance was determined using two-way ANOVA followed by Tukey’s post hoc test (*p* < 0.05).

As illustrated in Figure 1a–c, microwave-assisted extraction (MAE) exhibited a significant (*p* < 0.05) effect on the protein content of black gram, white pea, and red gram, depending on microwave power and irradiation time. The protein contents of the alkaline-extracted (native) protein powder were 71.11 ± 0.40% for red gram, 68.74 ± 0.32% for black gram, and 72.26 ± 0.32% for white pea protein, respectively. For red gram, protein content increased significantly from 71.11 ± 0.40% (native) to 74.92 ± 0.021% at 720 W for 60 s, followed by a decline at longer exposure times (120 s, 73.88 ± 0.057%). Similarly, in black gram protein, protein content increased from 73.87 ± 0.012% at 720 W for 60 s. A similar trend was observed for white pea protein, where protein content increased from 72.26 ± 0.32% to 76.32 ± 0.023% under the same extraction conditions, representing the highest yield among the three legumes. The observed improvement in protein recovery under MAE can be attributed to the rapid and uniform thermal energy input generated during microwave processing, which promotes matrix swelling, cell wall disruption, and enhanced solvent penetration, thereby facilitating protein solubilization and mass transfer into the extraction medium [39]. However, prolonged exposure or excessive energy input may lead to protein aggregation or partial denaturation, reducing extractability and resulting in yield plateauing or decline at extended treatment times. These findings are consistent with those of Coutinho et al. [40], who reported an increase in protein yield from pigeon pea using microwave-assisted extraction compared to conventional alkaline extraction. In contrast, Prandi et al. [41] observed lower protein recovery during MAE of green coffee bean proteins compared to ultrasound-assisted extraction (UAE), which was attributed to the higher thermal load associated with microwave processing. UAE operates at comparatively lower temperatures and relies primarily on cavitation-driven matrix disruption, which may better preserve protein integrity under certain conditions.

Overall, MAE significantly enhanced protein content across all three legumes, with optimal extraction consistently achieved at 720 W for 60 s. Although extraction time influenced protein recovery, microwave power exerted a more pronounced effect, as higher power levels intensified energy input and promoted efficient matrix disruption and protein solubilization [8,9]. Extending exposure beyond 60 s did not further improve protein yield and likely promoted aggregation or partial denaturation, thereby limiting extractability.

Based on these results, the MAE condition of 720 W for 60 s was selected as the optimal protocol for protein extraction from black gram, white pea, and red gram. Protein extracts obtained under this MAE condition were used for all subsequent structural, functional, and application-related analyses.

### 3.3. Characterization of Protein

#### 3.3.1. Amino Acid Content

**Figure 2:**
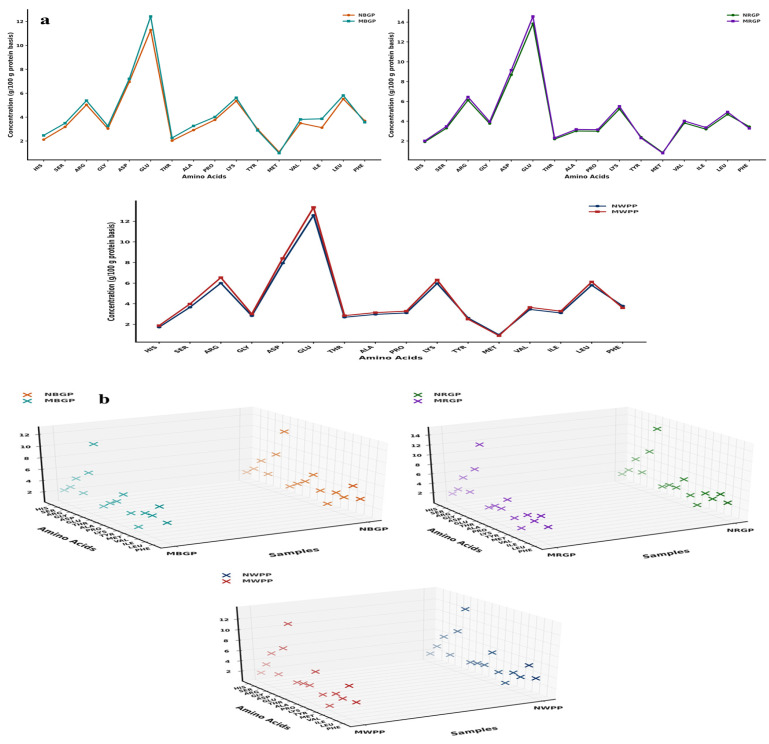

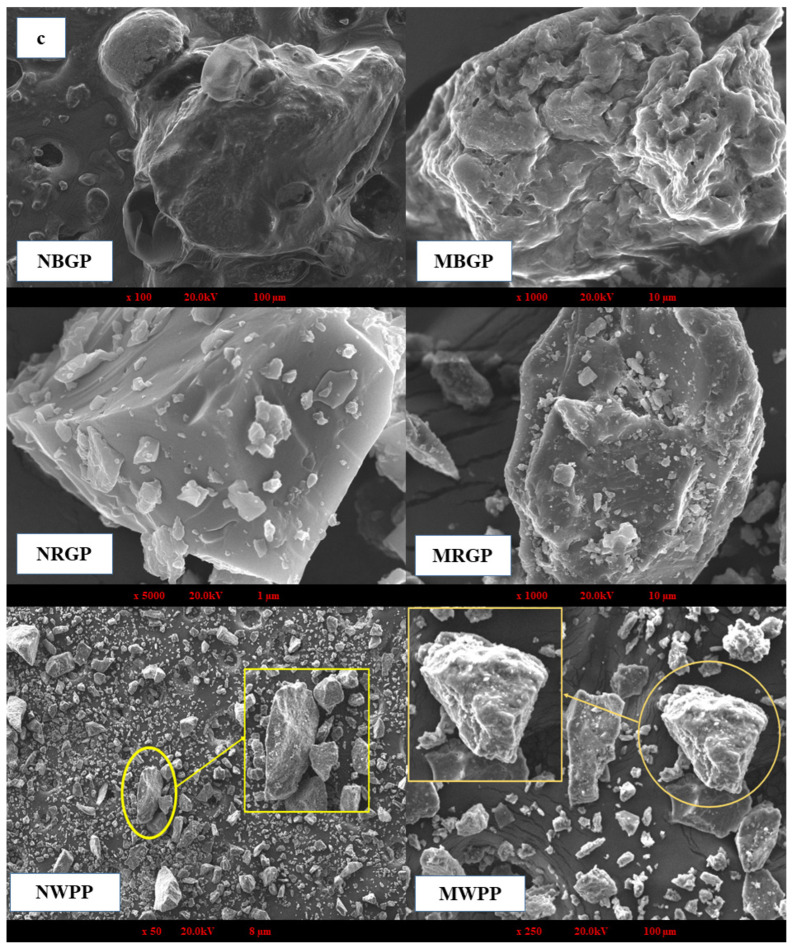

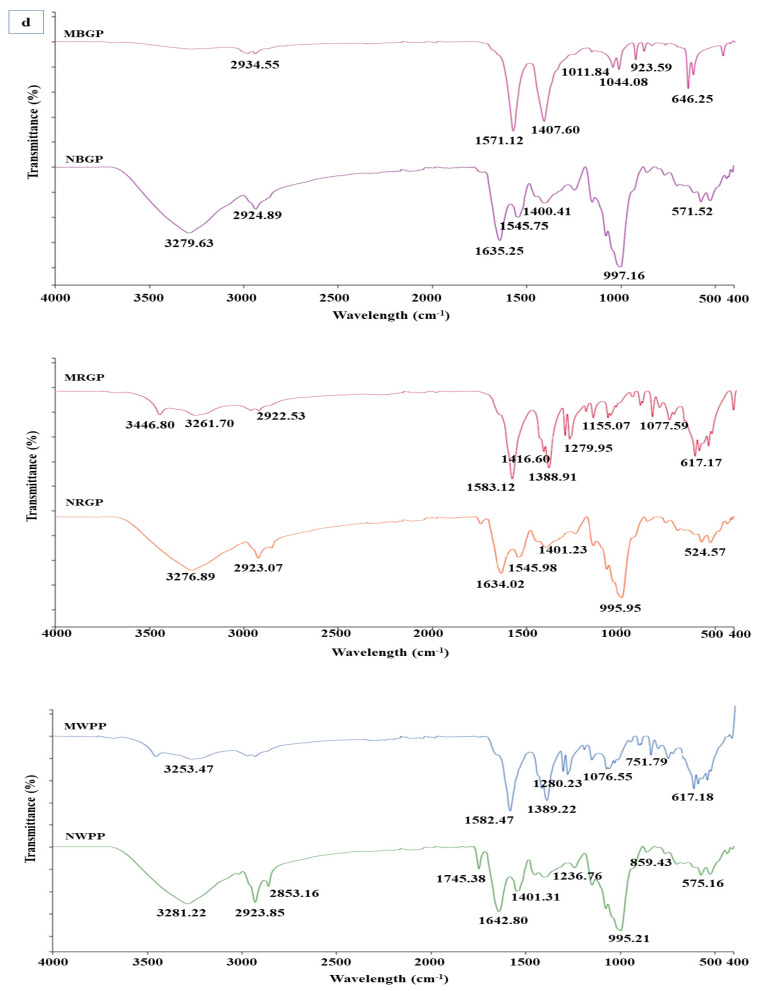

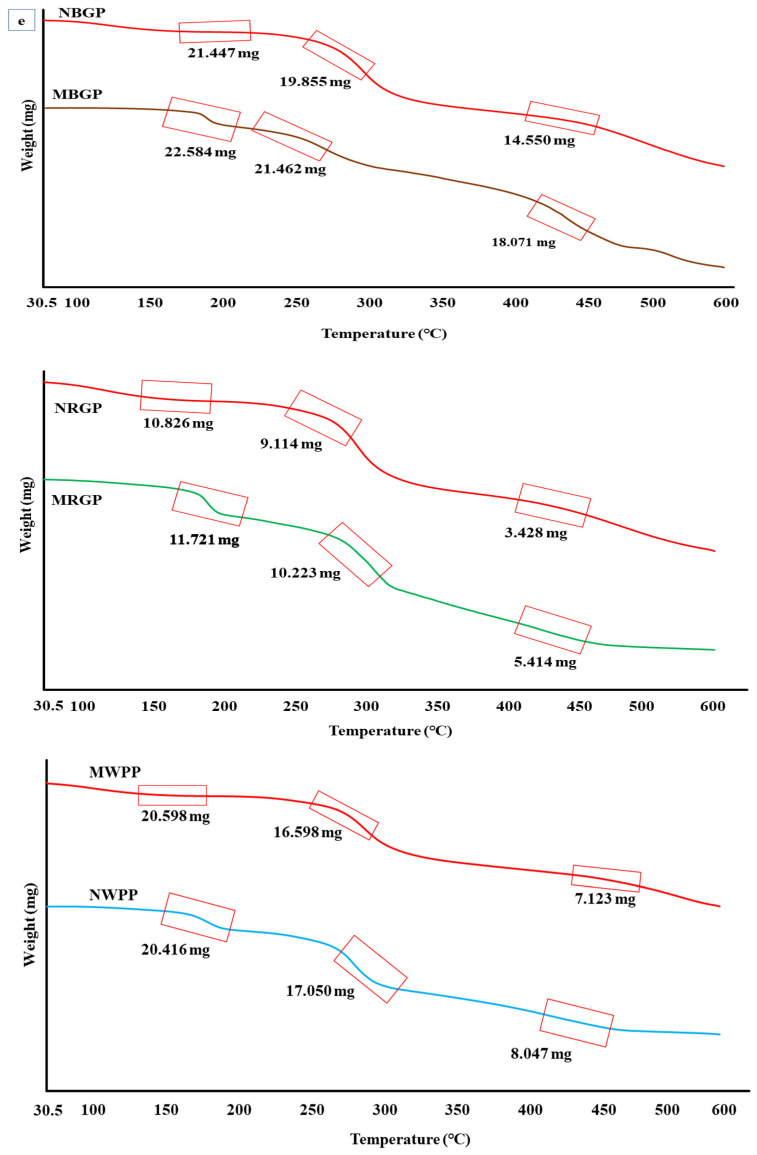
(**a**,**b**) Amino acid content, (**c**) scanning electron microscope, (**d**) Fourier transform infrared spectroscopy, and (**e**) thermogravimetric analysis of native and microwave-treated red gram (NRGP and MRGP), black gram (NBGP and MBGP), and white pea (NWPP and MWPP) proteins.

Amino acids are the fundamental building blocks of proteins, linked together through peptide bonds to form specific sequences that determine the protein’s structure, functionality, and nutritional quality. As shown in Figure 2a,b, the amino acid composition of black gram, white pea, and red gram proteins exhibited significant changes after microwave-assisted extraction (MAE) compared to native protein. These changes involved both increases and slight decreases in specific amino acids, reflecting differences in thermal stability, structural location, and accessibility of residues during microwave treatment. In black gram protein, hydrophilic amino acids such as aspartic acid increased from 6.94 to 7.2 g/100 g protein powder, and glutamic acid rose from 11.29 to 12.42 g/100 g. Increments were also observed for serine (3.18 to 3.49 g/100 g), histidine (2.12 to 2.47 g/100 g), arginine (5.03 to 5.38 g/100 g), proline (3.76 to 4.01 g/100 g), valine (3.49 to 3.81 g/100 g), isoleucine (3.12 to 3.86 g/100 g), and leucine (5.52 to 5.81 g/100 g). Minor decreases were observed in tyrosine (2.99 to 2.89 g/100 g) and phenylalanine (3.69 to 3.57 g/100 g) and the sulfur-containing methionine (1.08 to 0.99 g/100 g), likely due to slight thermal degradation. In contrast, branched-chain amino acids (BCAAs), valine (3.49 to 3.81 g/100 g), isoleucine (3.12 to 3.86 g/100 g), and leucine (5.52 to 5.81 g/100 g) increased, reflecting their relative heat stability and enhanced extractability. In white pea, a similar trend was observed. Acidic amino acids aspartic acid (7.93 to 8.4 g/100 g) and glutamic acid (12.59 to 13.35 g/100 g) increased, alongside serine (3.65 to 3.98 g/100 g) and arginine (6.003 to 6.54 g/100 g), suggesting improved accessibility of polar residues due to cell wall disruption. Proline showed a notable increase (3.10 to 3.28 g/100 g), indicative of significant protein unfolding. Minor decreases in tyrosine (2.62 to 2.50 g/100 g), phenylalanine (3.79 to 3.60 g/100 g), and methionine (0.99 to 0.90 g/100 g) were attributed to partial thermal sensitivity. BCAAs valine (3.45 to 3.65 g/100 g), isoleucine (3.10 to 3.28 g/100 g), and leucine (5.79 to 6.12 g/100 g) demonstrated enhanced recovery, highlighting the efficiency of MAE in preserving essential amino acids. For red gram, increases in aspartic acid (8.69 to 9.15), glutamic acid (13.85 to 14.57 g/100 g), serine (3.29 to 3.47 g/100 g), and arginine (6.12 to 6.44) reflected the effective disruption of protein matrices. Glycine (3.76 to 3.95 g/100 g) and proline (2.99 to 3.15 g/100 g) also increased due to exposure of internal residues. Minor reductions in tyrosine (2.39 to 2.30 g/100 g), phenylalanine (3.45 to 3.28 g/100 g), and methionine (0.83 to 0.78 g/100 g) occurred, likely due to heat-labile degradation. BCAAs such as valine (3.82 to 4.02 g/100 g), isoleucine (3.18 to 3.36 g/100 g), and leucine (4.69 to 4.93 g/100 g) were effectively recovered, showing good thermal stability. Similarly, as shown in Figure 2b, the 3D scatter plots clearly present the changes in amino acid composition between native and microwave-assisted extracted (MAE) proteins. Each plot compares paired protein samples NWPP (native white pea protein) and MWPP (microwave assisted extracted white pea protein), NRGP (native red gram protein) and MRGP (microwave assisted extracted red gram protein), and NBGP (native black gram protein) and MBGP (microwave assisted black gram protein) by placing amino acids on the *Y*-axis, sample codes on the *X*-axis, and amino acid concentrations (g/100 g protein) on the *Z*-axis. Different color codes separate the native (N) and modified (M) proteins, and the tilted *Y*-axis helps to display all amino acid names clearly. The 3D view highlights the concentration differences between the paired samples, showing that modified proteins have higher amino acid concentrations than native ones. Overall, these 3D visualizations support the compositional data by providing a clear spatial representation of how MAE affects the amino acid composition of legume proteins.

Overall, MAE resulted in a higher total amino acid content across all legumes compared to native proteins. Total amino acids increased from 65.56 to 70.30 g/100 g protein in black gram, from 69.23 to 72.78 g/100 g protein in white pea, and from 69.41 to 72.39 g/100 g protein in red gram. These results indicate enhanced protein recovery and improved extraction efficiency rather than chemical modification of amino acids. Our study aligns with the findings of Mali et al. [42], who reported an increase in amino acid content of black bean protein following microwave-assisted extraction compared to conventional alkaline extraction. In the present study, the total amino acid content of black bean protein increased from 80.46 in the control (alkaline extraction) to 85.97 after microwave-assisted extraction, indicating enhanced protein quality and recovery.

#### 3.3.2. Scanning Electron Microscope

Scanning electron microscopy (SEM) is a high-resolution imaging technique used to examine the surface morphology and microstructural features of protein powders. It provides detailed insights into the topographical and structural changes that occur during processing, such as aggregation, unfolding, and surface porosity, which directly influence solubility, functionality, and interaction behavior of proteins in food systems. As shown in Figure 2c, distinct morphological variations were observed between the native and microwave-assisted protein samples of black gram protein (BGP), red gram protein (RGP), and white pea protein (WPP). Native black gram protein exhibited compact, irregular agglomerates with limited surface porosity, indicating a dense protein matrix. Similarly, native red gram protein showed relatively smooth and crystalline-like surfaces, reflecting a tightly packed structure. Native white pea protein displayed large, smooth flakes with minimal surface irregularities. In contrast, MAE-treated proteins from all three legumes exhibited fractured, rough, and fragmented particles with increased surface irregularities and reduced compactness. These morphological changes suggest enhanced matrix disruption and partial structural loosening under MAE conditions, which may facilitate protein release and solubilization during extraction. The observed increase in surface roughness and porosity is consistent with improved extraction efficiency and functional performance reported in subsequent analyses. A similar trend was observed in the study of Mali et al. [42], who found that microwave treatment reduced aggregation in black bean protein isolates. The untreated samples exhibited irregular, rough surfaces due to hydrophobic and hydrogen-bonded interactions, whereas microwave irradiation enhanced molecular repulsion, weakened aggregates, and produced a more uniform morphology.

#### 3.3.3. Fourier Infrared Spectroscopy

Fourier Transform Infrared Spectroscopy (FTIR) is a powerful analytical technique used to identify the functional groups and study the secondary structure of proteins through characteristic vibrational frequencies of chemical bonds. As shown in Figure 2d, distinct spectral variations were observed between native and microwave-assisted protein concentrates of black gram (BGP), red gram (RGP), and white pea (WPP), indicating structural modifications in the protein backbone and side chains. For black gram protein (NBGP), the native sample exhibited strong absorption peaks at 3279.63 cm^−1^ and 2924.89 cm^−1^, corresponding to N–H stretching of amide A and C–H stretching vibrations, respectively. The amide I and II bands appeared around 1635.25 cm^−1^ and 1545.75 cm^−1^, representing C=O stretching and N–H bending. After microwave-assisted extraction (MBGP), these bands shifted to 2934.55 cm^−1^, 1571.12 cm^−1^, and 1407.60 cm^−1^, suggesting partial unfolding of peptide bonds and formation of new hydrogen bonds. The appearance of sharper peaks at 1011.84 to 1044.08 cm^−1^ reflects enhanced exposure of C–N and C–O stretching vibrations due to rearrangement of protein matrix. Similarly, native red gram protein (NRGP) exhibited peaks at 3276.89 cm^−1^ and 2923.07 cm^−1^ for N–H and C–H stretching, and amide I and II bands at 1634.02 cm^−1^ and 1545.98 cm^−1^. After microwave treatment (MRGP), these shifted to 3446.80 cm^−1^, 3261.70 cm^−1^, and 1583.12 cm^−1^, respectively. The shift and broadening of amide I and II bands indicate an alteration in secondary structure. New peaks at 1279.95 and 1155.07 cm^−1^ correspond to C–O and C–N stretching, implying formation of new polar interactions. In the case of white pea protein (NWPP), major peaks at 3281.22 cm^−1^ (N–H stretching), 2923.85 cm^−1^ (C–H stretching), and 1642.80 cm^−1^ (amide I) confirmed the presence of ordered α-helices and β-sheets in the native form. After microwave-assisted extraction (MWPP), these bands slightly shifted to 3253.47 cm^−1^ and 1582.47 cm^−1^, suggesting rearrangement of hydrogen bonding and transition of some α-helical regions into random coils. Additional bands observed between 1076.55 and 1280.23 cm^−1^ indicate increased exposure of polar side chains due to molecular unfolding.

Overall, FTIR spectra confirmed that microwave-assisted extraction induced moderate conformational changes in legume proteins, leading to partial unfolding and reorganization of secondary structures. The study by Mali et al. [42] showed that the microwave treatment of black bean protein altered the secondary structure of protein, where characteristic peaks of amide A (3264 cm^−1^), amide I (1635 cm^−1^), amide II (1537 cm^−1^), and glycoprotein region (1053 cm^−1^) showed slight shifts under different microwave power levels.

#### 3.3.4. Thermogravimetric Analysis

Thermogravimetric analysis (TGA) is a thermal analytical technique that measures the thermal stability, degradation behavior, and compositional characteristics of biomolecules such as proteins. As shown in Figure 2e, the TGA curves of native and microwave-assisted protein concentrates from black gram (BGP), red gram (RGP), and white pea (WPP) demonstrated distinct weight loss patterns, indicating differences in their thermal stability and structural integrity. For black gram protein, the native sample (NBGP) exhibited an initial weight of 21.869 mg, which decreased gradually with temperature, leaving 21.447 mg at 150 °C, 19.855 mg at 250 °C, and 14.550 mg at 450 °C. This three-step weight reduction represents moisture evaporation and a decrease in volatile components (up to 150 °C), denaturation of protein fractions (200–300 °C), and degradation of peptide bonds and amino acid side chains (above 350 °C) [42,43,44]. In contrast, the microwave-assisted extract (MBGP) with an initial weight of 22.910 mg retained 22.584 mg at 150 °C, 21.462 mg at 250 °C, and 18.071 mg at 450 °C, showing comparatively lower weight loss. In the case of red gram protein, the native sample (NRGP) decreased from 11.793 mg to 10.826 mg at 150 °C, 9.114 mg at 250 °C, and 3.428 mg at 450 °C. In comparison, the microwave-treated sample (MRGP) exhibited higher mass retention (11.721 mg, 10.223 mg, and 5.414 mg at the same temperatures), confirming greater thermal resistance. Similarly, for white pea protein, the native extract (NWPP) decreased from 21.116 mg to 20.416 mg at 150 °C, 17.050 mg at 250 °C, and 8.047 mg at 450 °C, whereas the microwave-assisted sample (MWPP) retained slightly more mass (20.598 mg, 16.598 mg, and 7.123 mg), suggesting slightly enhanced thermal stability. Overall, TGA revealed consistently higher thermal stability in microwave-assisted protein extracts, a trend also reported by Mali et al. [42] for microwave-treated black bean protein.

### 3.4. Functional Properties of Protein

#### 3.4.1. Water- and Oil-Holding Capacity (WHC and OHC)

**Figure 3:**
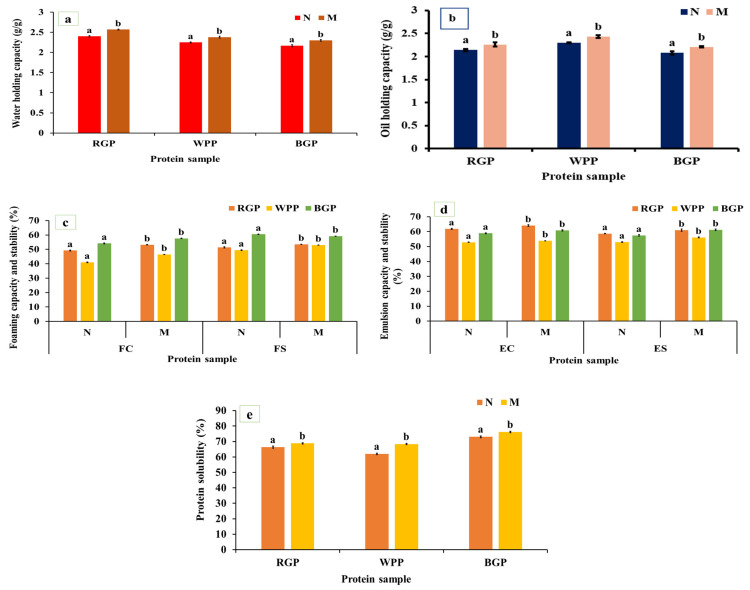
(**a**) Water-holding capacity, (**b**) oil-holding capacity, (**c**) foaming capacity and stability, (**d**) emulsion capacity and stability, and (**e**) protein solubility of native and microwave-treated red gram (NRGP and MRGP), black gram (NBGP and MBGP), and white pea (NWPP and MWPP) proteins. The results are expressed as the mean ± standard deviation of ≥3 independent replicates, and error bars represent the standard deviation from the mean values. Different lowercase letters above each bar represent significantly different values at *p* < 0.05 according to an independent samples *t*-test.

WHC and OHC represent the ability of proteins to bind and retain water or oil, respectively, and are important in determining the texture, mouthfeel, and stability of food matrices such as doughs, batters, and emulsions. As shown in Figure 3a,b, both WHC and OHC were significantly increased (*p* < 0.05) in microwave-assisted extracted (MAE) proteins compared to native proteins for all legumes. In red gram protein, WHC increased from 2.40 to 2.57 g/g and OHC from 2.14 to 2.26 g/g. In white pea, WHC improved from 2.25 to 2.38 g/g and OHC from 2.30 to 2.43 g/g. In black gram protein, WHC increased from 2.17 to 2.30 g/g and OHC from 2.08 to 2.21 g/g. The significant improvement in WHC and OHC under MAE can be attributed to microwave-induced heating, which rapidly disrupts hydrogen bonds, unfolds protein molecules, and exposes more hydrophobic groups [40,45,46]. The increased exposure of hydrophilic residues such as aspartic acid, glutamic acid, and serine, as shown in Figure 2a, improves water retention through hydrogen bonding. Similarly, the exposure of hydrophobic amino acids such as leucine, isoleucine, and valine facilitates oil binding through hydrophobic interactions. In MAE-treated samples, the partial unfolding and loosening of protein structure, confirmed by SEM micrographs showing a more porous morphology, allow for greater interfacial area for molecular interactions with water and oil. Similar observations were made by Coutinho et al. [40], where the WHC and OHC of microwave-treated pea protein rose by 13.93% and 20%, respectively, compared to the untreated protein.

#### 3.4.2. Foaming Capacity and Stability

Foaming capacity (FC) represents the ability of proteins to form stable air–water interfaces during whipping or blending, whereas foaming stability (FS) reflects the capacity of these foams to resist collapse over time. As shown in Figure 3c, both FC and FS significantly improved (*p* < 0.05) after microwave-assisted extraction (MAE) compared to the native protein. Red gram protein exhibited an increase from 49.19% to 53.16% (FC) and 51.36% to 53.54% (FS), while white pea protein improved from 41.02% to 46.51% (FC) and 49.52% to 52.98% (FS). In black gram protein, FC increased from 54.17% to 57.62% and FS from 60.47% to 62.95%. The observed enhancement in foaming properties is likely associated with increased protein flexibility and improved interfacial activity resulting from structural rearrangements induced during MAE processing [47,48]. The amino acid profiling data revealed notable increases in surface-active amino acids as shown in Figure 2a. Furthermore, the partial loosening of protein aggregates due to MAE may facilitate faster adsorption and more uniform distribution of proteins at the air–water interface, promoting foam formation and stabilization [45]. Our results are comparable to the findings of Kadam et al. [46], who reported an increase in foaming capacity (FC) of microwave-treated cottonseed protein. However, unlike our observations, their study showed a decline in foaming stability (FS) following microwave treatment.

#### 3.4.3. Emulsion Capacity and Stability

Emulsion capacity (EC) defines the ability of proteins to adsorb at the oil–water interface during emulsification, while emulsion stability (ES) refers to the resistance of these emulsions to coalescence or phase separation over time. Both properties depend on the interfacial behavior, flexibility, solubility, and amphiphilic balance of the protein molecules. As illustrated in Figure 3d, the EC and ES of all legume proteins increased significantly (*p* < 0.05) after microwave-assisted extraction (MAE) compared to the native samples. In red gram, EC rose from 61.80% to 64.03%, while ES improved from 58.66% to 61.95%. Similarly, white pea protein exhibited increases from 55.07% to 57.52% (EC) and 54.51% to 56.39% (ES). For black gram protein, EC increased from 61.80% to 64.03%, and ES from 58.66% to 60.95%. These improvements can be attributed to enhanced interfacial behavior of proteins following MAE, including improved dispersion, increased structural flexibility, and more effective adsorption at the oil–water interface [48,49]. The SEM micrographs further confirm a more fragmented and porous structure in MAE-treated proteins, providing increased interfacial surface area and better adsorption capacity. Similar improvements were also described by Shi et al. [48], who noted that microwave treatment improved the functional attributes of *Lactarius volemus* protein, resulting in increases of emulsifying capacity (EC) and emulsifying stability (ES) compared to the water bath assisted extraction.

#### 3.4.4. Protein Solubility

Protein solubility represents the extent to which protein molecules disperse in an aqueous medium, primarily governed by their surface polarity, charge distribution, molecular flexibility, and hydrophobic–hydrophilic balance. It is one of the most critical determinants of functional behavior, influencing emulsification, foaming, and gelation capacities. As represented in Figure 3e, microwave-assisted extracted (MAE) proteins exhibited markedly higher (*p* < 0.05) solubility compared to native proteins across all legumes. For red gram, solubility increased from 67.25% to 70.84%, and in white pea protein, from 65.47% to 68.12%, and black gram protein, from 68.41% (native) to 72.15% (MAE). The increased solubility may be attributed to microwave heating, which promotes the formation of hydrogen bonds between protein molecules and water, thereby enhancing protein–water interactions. In addition, microwave heating can induce conformational changes that expose hydrophilic groups, further contributing to improved solubility [50]. The amino acid profile supports this observation, showing elevated levels of acidic (Asp, Glu) and basic (Lys, Arg) residues in MAE samples. Tang et al. [51] observed that applying microwave treatment led to an increase in the solubility of lupin (*Lupinus angustifolius* L.) protein.

Microwave-assisted extraction (MAE) significantly enhanced the functional properties of all legume proteins compared to native forms, demonstrating improved solubility, WHC, OHC, foaming, and emulsifying abilities compared to native proteins. These enhancements are associated with structural loosening, improved dispersion, and altered interfacial behavior induced during MAE processing, as supported by SEM and FTIR analyses. Among the studied legumes, black gram protein exhibited the highest improvement, followed by red gram and white pea. Overall, MAE proved to be a rapid and efficient technique for producing functionally superior legume protein.

### 3.5. Bioactive Compounds in Protein

#### 3.5.1. Total Phenolic and Flavonoid Content

**Figure 4:**
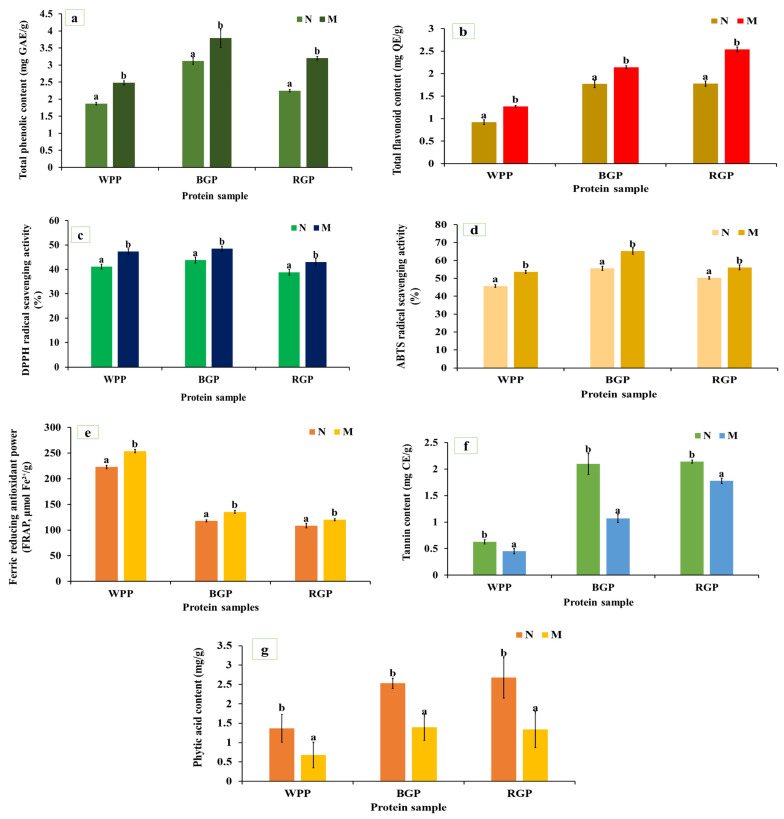
(**a**) Total phenolic content (TPC), (**b**) total flavonoid content (TFC), (**c**) DPPH radical scavenging activity, (**d**) ABTS radical scavenging activity, (**e**) ferric-reducing antioxidant power (FRAP), (**f**) tannin content, and (**g**) phytic acid content of native and microwave-treated red gram (NRGP and MRGP), black gram (NBGP and MBGP), and white pea (NWPP and MWPP) proteins. The results are expressed as the mean ± standard deviation of ≥3 independent replicates, and error bars represent the standard deviation from the mean values. Different lowercase letters above each bar represent significantly different values at *p* < 0.05 according to an independent samples *t*-test.

Total phenolic content (TPC) shows the concentration of phenolic compounds that can donate hydrogen atoms or electrons to neutralize free radicals, thereby contributing to the antioxidant potential of legume proteins. As shown in Figure 4a, a significant (*p* < 0.05) increase in TPC was observed in all three legumes after microwave-assisted extraction (MAE) compared to their native protein. The TPC of white pea increased from 1.87 to 2.48 mg GAE/g, black gram from 3.12 to 3.79 mg GAE/g, and red gram from 2.25 to 3.20 mg GAE/g. Similarly, as represented in Figure 4b, the total flavonoid content (TFC) also increased significantly (*p* < 0.05) after MAE, from 0.92 to 1.27 mg QE/g in white pea, 1.78 to 2.54 mg QE/g in black gram, and 1.77 to 2.14 mg QE/g in red gram. The enhancement in TPC and TFC following MAE can be attributed to the combined effects of thermal energy and improved mass transfer during extraction [52].

#### 3.5.2. DPPH Radical Scavenging Activity

The DPPH assay measures the hydrogen-donating ability of bioactive compounds, reflecting their antioxidant strength. As illustrated in Figure 4c, all MAE-extracted protein samples displayed significantly higher (*p* < 0.05) DPPH scavenging activity than their native protein. White pea protein showed a significant increase, increasing from 41.11 to 47.29%, followed by black gram (from 43.81 to 48.45%) and red gram (from 38.82 to 43.01%). The improved DPPH activity is consistent with higher phenolic and flavonoid contents observed after MAE. MAE promotes heating, leading to cell wall disruption and enhanced release of phenolic and other compounds responsible for DPPH radical scavenging activity [53]. In addition, amino acid data (Figure 1a) confirms these trends, showing clear increases in histidine (1.725 to 1.88 g/100 g in WPP, 1.91 to 2.01 g/100 g in RGP, and 2.12 to 2.47 g/100 g in BGP) and serine (3.657 to 3.98 g/100 g in WPP, 3.72 to 4.03 g/100 g in RGP, and 3.58 to 3.89 g/100 g in BGP), together with improved accessibility of other abundant residues, across all three legumes following MAE. The comparatively greater increase observed in white pea protein may be related to its less compact protein structure, allowing for more extensive conformational changes during extraction and improved exposure of antioxidant active sites.

#### 3.5.3. ABTS Radical Scavenging Activity

The ABTS assay measures the ability of antioxidants to quench the ABTS^+^ radical cation through electron or hydrogen donation, reflecting the total antioxidant potential of proteins. As shown in Figure 4d, microwave-assisted extraction (MAE) caused a significant increase (*p* < 0.05) in ABTS scavenging activity in all three legume proteins. For white pea, activity increased from 45.7 to 53.67%; for black gram, from 55.60 to 65.29%; and from 50.24 to 56.11% for red gram. The enhanced ABTS activity is closely related to the enhanced TPC and TFC obtained after MAE, indicating that the improved antioxidant capacity is primarily associated with the greater availability of phenolic and other electron-donating compounds released during extraction [54,55,56].

#### 3.5.4. Ferric Reducing Power Assay

Ferric reducing power reflects the electron-donating potential of antioxidants capable of reducing Fe^3+^ to Fe^2+^. As shown in Figure 4e, the FRAP values of MAE-extracted samples were significantly higher (*p* < 0.05) compared to native proteins. For white pea, the FRAP increased from 222.92 to 253.74 μmol Fe^2+^/g, for black gram from 117.62 to 135.28 μmol Fe^2+^/g, and for red gram from 108.39 to 135.28 μmol Fe^2+^/g. The enhanced ferric reducing power is attributed to improved extraction of redox-active compounds under MAE conditions, consistent with the increases observed in phenolic and flavonoid contents [57,58,59].

#### 3.5.5. Tannin and Phytic Acid Content

As shown in Figure 4f,g, MAE significantly reduced (*p* < 0.05) tannin and phytic acid levels across all legumes. White pea tannin content decreased from 0.63 to 0.45 mg TAE/g, black gram from 2.1 to 1.07 mg TAE/g, and red gram from 2.14 to 1.78 mg TAE/g. Similarly, phytic acid content decreased from 1.37 to 0.68 mg/g (white pea), 2.68 to 1.34 mg/g (red gram), and 2.53 to 1.4 mg/g (black gram). The reduction in tannin and phytic acid after MAE indicates the sensitivity of these antinutritional factors to thermal processing. Heat-induced degradation or structural modification of these compounds during extraction likely contributed to their lower measurable levels [60].

Microwave-assisted extraction (MAE) showed a significant improvement in the release and activity of bioactive compounds across all three legumes, but a significant decrease in tannin and phytic acid contents was also observed in all legumes. Overall, the microwave process exerted a positive impact on the antioxidant and nutritional potential of all legumes, especially white pea and red gram, while black gram showed moderate enhancement.

### 3.6. In Vitro Digestibility of Protein

**Figure 5:**
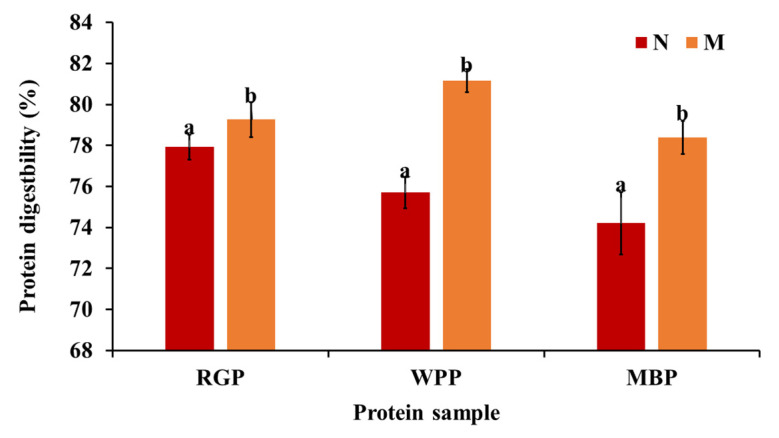
Protein digestibility of native and microwave-treated red gram (NRGP and MRGP), black gram (NBGP and MBGP), and white pea (NWPP and MWPP) proteins. The results were expressed as the mean ± standard deviation of ≥3 independent replicates, and error bars represent the standard deviation from the mean values, while different lowercase letters above each bar represent significantly different values. The results are expressed as the mean ± standard deviation of ≥3 independent replicates, and error bars represent the standard deviation from the mean values. Different lowercase letters above each bar represent significantly different values at *p* < 0.05 according to an independent samples *t*-test.

Protein digestibility represents the extent to which protein structures are hydrolyzed by digestive enzymes, determining the bioavailability of amino acids and peptides. As shown in Figure 5, microwave-assisted extraction significantly enhanced (*p* < 0.05) the digestibility of all legume proteins. The digestibility of red gram increased from 77.92 to 79.26%, white pea from 75.70 to 81.16%, and black gram from 74.21 to 78.4%. The improvement in digestibility after microwave-assisted extraction (MAE) can be explained by structural changes induced in proteins and the reduction in antinutritional factors, as explained above. Microwave treatment has been shown to promote partial unfolding and denaturation of proteins, exposing hydrophobic residues and increasing enzyme accessibility, which enhances in vitro protein digestibility by facilitating enzymatic hydrolysis [61,62]. The SEM results support this, showing a more porous and fragmented surface morphology in microwave-treated samples, which allows for better enzyme penetration. Furthermore, the amino acid profile data reveal an increase in total amino acid content, particularly in aspartic acid, glutamic acid, and lysine residues that enhance solubility and enzyme substrate interaction. The higher solubility observed in functional property analysis further supports this finding, as soluble proteins are more susceptible to enzymatic hydrolysis. Our findings are relatable to the research by Mali et al. [42], in which, after microwave treatment, the black bean protein digestibility increased compared to the untreated sample.

Overall, MAE led to a consistent enhancement in protein digestibility across all legumes, reflecting improved enzymatic accessibility rather than microwave-specific effects. Among the three legumes, white pea protein exhibited the highest absolute digestibility after extraction, while black gram showed a comparatively greater relative improvement from its native state.

### 3.7. Protein Fortified Flour

#### 3.7.1. Proximate Analysis of the Protein Powder

**Figure 6:**
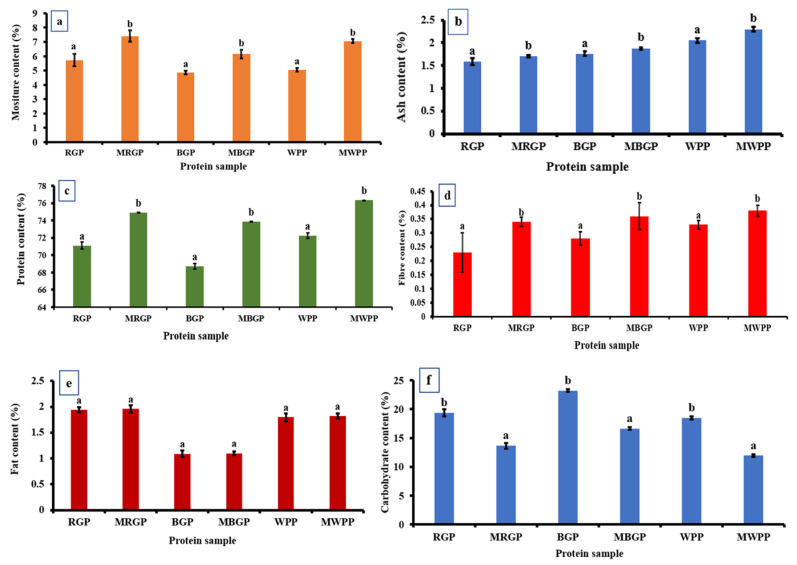
Proximate analysis of protein powder (**a**) moisture, (**b**) ash, (**c**) protein, (**d**) fiber, (**e**) fat, and (**f**) carbohydrate contents of native and microwave-treated red gram (NRGP and MRGP), black gram (NBGP and MBGP), and white pea (NWPP and MWPP) proteins. The results are expressed as the mean ± standard deviation of ≥3 independent replicates, and error bars represent the standard deviation from the mean values. Different lowercase letters above each bar represent significantly different values at *p* < 0.05 according to an independent samples *t*-test.

As shown in Figure 6, the proximate composition of native and microwave-treated legume protein showed clear shifts across all components. Moisture content (Figure 6a) increased sharply after microwave treatment in all samples, rising from 5.73% to 7.40% in red gram, 4.86% to 6.15% in black gram, and 5.06% to 7.06% in white pea protein, indicating enhanced water-binding capacity. Ash content also increased consistently, with RGP increasing from 1.59% to 1.71%, BGP from 1.76% to 1.88%, and WPP from 2.05% to 2.30%, reflecting a higher concentration of mineral residues as illustrated in Figure 6b. Protein content showed (Figure 6c) a significant improvement in microwave-treated samples, increasing from 71.11% to 74.92% in red gram, 68.74% to 73.87% in black gram, and 72.26% to 76.32% in white pea protein, largely due to moisture reduction and structural concentration effects. As represented in Figure 6d, fiber content also increased slightly in all treated samples. In contrast, fat content remained unaffected, with non-significant differences between native and treated samples as shown in Figure 6e. Carbohydrate content decreased after microwave treatment across all legumes, dropping from 19.38% to 13.67% in red gram, 23.24% to 16.66% in black gram, and 18.49% to 11.96% in white pea protein, consistent with a relative increase in protein and moisture fractions (Figure 6f).

#### 3.7.2. Nutritional Properties of Flour

**Figure 7:**
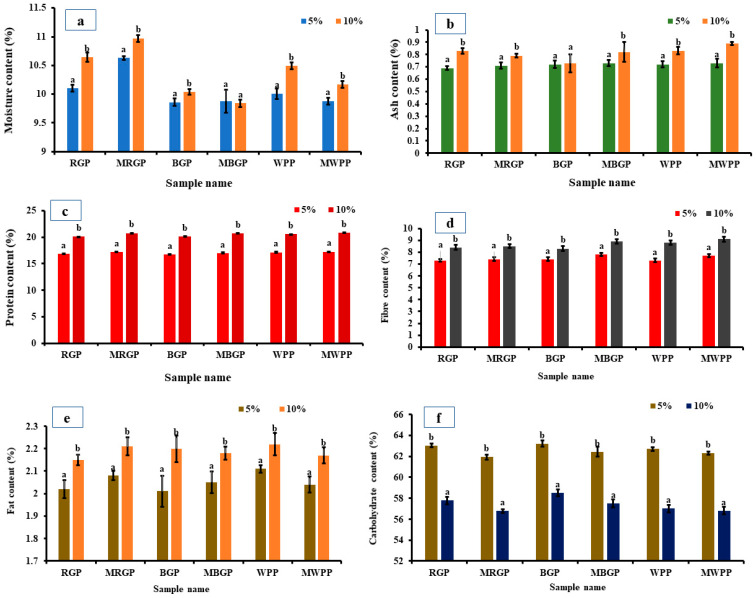
(**a**) Moisture, (**b**) ash, (**c**) protein, (**d**) fiber, (**e**) fat, and (**f**) carbohydrate contents of native and microwave-treated red gram (NRGP and MRGP), black gram (NBGP and MBGP), and white pea (NWPP and MWPP) proteins incorporated (5 and 10%) in wheat flour. The results are expressed as the mean ± standard deviation of ≥3 independent replicates, and error bars represent the standard deviation from the mean values. Different lowercase letters above each bar represent significantly different values at *p* < 0.05 according to an independent samples *t*-test.

The nutritional properties of wheat flour blended with 5% and 10% native and microwave-treated legume protein concentrates are presented in Figure 7. The moisture content of the blend (Figure 7a) increased significantly (*p* < 0.05) at 10% protein incorporation, with microwave-treated samples (MRGP, MBGP, and MWPP) showing slightly higher values than the blends containing native proteins. Ash content (Figure 7b) also increased in the 10% blends, particularly in microwave-treated proteins, indicating better mineral retention. Protein content (Figure 7c) rose significantly with the incorporation level of 10% blends, with microwave-treated proteins contributing more due to enhanced solubility and extractability. Fiber (Figure 7d) and fat contents (Figure 7e) showed significant increases at higher incorporation levels, again with microwave-treated proteins showing slightly higher values, likely due to the disruption of fiber–protein and lipid–protein interactions during microwave processing. Conversely, carbohydrate content (Figure 7f) decreased significantly in 10% blends as a dilution effect, given the replacement of carbohydrate-rich wheat flour with protein-rich legume concentrate.

#### 3.7.3. Water- and Oil-Holding Capacity of Flour

The water- and oil-holding capacities of wheat flour incorporated with 5% and 10% native and microwave-treated legume protein concentrate are presented in Figure 7a. Both WHC and OHC increased significantly (*p* < 0.05) with higher incorporation levels, with the 10% blends consistently showing superior capacities compared to the 5% blends. Blends containing microwave-treated proteins exhibited slightly higher WHC and OHC compared to those containing native proteins. This behavior is consistent with the improved hydration and lipid interaction properties of the treated proteins observed in earlier functional analyses. Overall, the results confirm that protein fortification with flour enhances the functional performance of fortified wheat flour in terms of water and oil binding capacity.

#### 3.7.4. Water Solubility Index of Flour

**Figure 8:**
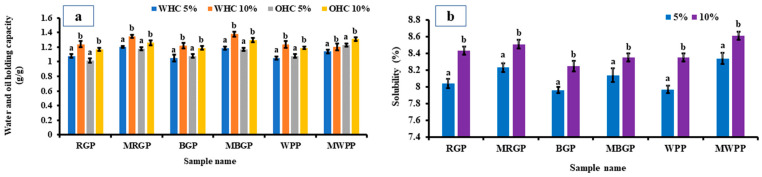
(**a**) Water- and oil-holding capacity, and (**b**) solubility of native and microwave-treated red gram (NRGP and MRGP), black gram (NBGP and MBGP), and white pea (NWPP and MWPP) protein incorporated (5 and 10%) wheat flour. The results were expressed as the mean ± standard deviation of ≥3 independent replicates, and error bars represent the standard deviation from the mean values, while different lowercase letters above each bar represent significantly different values.

The solubility index of wheat flour incorporated with 5% and 10% native and microwave-treated legume protein concentrate is illustrated in Figure 8a,b. Significant increases (*p* < 0.05) in solubility were observed at the 10% incorporation level for all samples, with microwave-treated proteins consistently exhibiting markedly higher solubility than their native proteins. The improved solubility of MAE-treated protein blends is consistent with their enhanced dispersion behavior observed in extracted protein systems. Improved solubility within the wheat flour matrix is advantageous for processing performance, as it can influence dough handling and hydration characteristics. These results demonstrate that microwave-assisted extraction produces protein ingredients that are more compatible with cereal-based systems without adversely affecting flour composition.

## 4. Conclusions

Microwave-assisted extraction (MAE) was shown to be an efficient and environmentally sustainable method for enhancing the yield, compositional attributes, and functional properties of legume proteins obtained from black gram, white pea, and red gram. Relative to native proteins, MAE significantly increased protein recovery, solubility, water- and oil-holding capacity, and emulsifying and foaming properties. These improvements were associated with alterations in protein secondary structure and surface morphology, as evidenced by FTIR and SEM analyses. MAE-treated proteins also exhibited improved thermal stability, increased availability of amino acids and phenolic compounds, reduced levels of antinutritional factors, and a moderate but statistically significant enhancement in in vitro protein digestibility. Furthermore, incorporation of MAE-derived proteins into wheat flour systems resulted in improved nutritional composition and functional performance, indicating their suitability for cereal-based food applications. However, the absence of temperature-matched conventional heating controls limits the ability to distinguish microwave-specific effects from general thermal effects. Future studies incorporating appropriate thermal controls are required to clarify the underlying mechanisms of protein modification during MAE. Overall, MAE constitutes a viable processing approach for the production of functionally improved legume protein ingredients for sustainable food applications.

## Figures and Tables

**Table 1:** Microwave-assisted extraction of red gram, black gram, and white pea protein at different powers and times.

Power (W)	Time (s)			
180	30 (34.67 °C)	60 (44.45 °C)	90 (56.81 °C)	120 (70.24 °C)
360	30 (42.56 °C)	60 (58.80 °C)	90 (74.15 °C)	120 (88.39 °C)
540	30 (50.34 °C)	60 (73.29 °C)	90 (85.48 °C)	120 (90.89 °C)
720	30 (58.73 °C)	60 (88.11 °C)	90 (93.21 °C)	120 (99.93 °C)

Sample temperatures were recorded immediately after microwave treatment using a calibrated digital thermometer.

**Table 2:** Proximate composition of red gram, white pea, and black gram powder.

Proximate Composition	Results (%)		
	Red Gram	White Pea	Black Gram
Moisture content	10.95 ± 0.35	7.56 ± 0.34	10.77 ± 0.10
Ash content	3.01 ± 0.04	3.02 ± 0.02	3.17 ± 0.03
Fat content	1.21 ± 0.03	2.72 ± 0.05	1.20 ± 0.06
Protein content	23.72 ± 0.40	24.74 ± 0.36	26.22 ± 0.31
Fiber content	6.24 ± 0.06	6.16 ± 0.05	5.11 ± 0.08
Carbohydrate content	54.87 ± 0.11	55.80 ± 0.21	53.53 ± 0.50

The carbohydrate content was calculated using the difference method. Data are presented as mean ± standard deviation (SD) of triplicate measurements.

## Data Availability

The original contributions presented in this study are included in the article. Further inquiries can be directed to the corresponding authors.
